# Increased BST2 expression during simian immunodeficiency virus infection is not a determinant of disease progression in rhesus monkeys

**DOI:** 10.1186/s12977-015-0219-8

**Published:** 2015-11-10

**Authors:** Bianka Mussil, Aneela Javed, Katharina Töpfer, Ulrike Sauermann, Sieghart Sopper

**Affiliations:** Unit of Infection Models, German Primate Centre, Goettingen, Germany; Department of Healthcare Biotechnology, Atta-ur-Rahman School of Applied Biosciences (ASAB), National University of Science and Technology (NUST), H12, Islamabad, Pakistan; Department of Hematology and Oncology, Medical University Innsbruck, ZVG 7G5 009A, Anichstr. 35, 6020 Innsbruck, Austria; Tyrolean Cancer Research Institute, Innsbruck, Austria

**Keywords:** BST2, Rhesus macaque, SIV, MX1, LTNPs, Real-time PCR, PBMC

## Abstract

**Background:**

Bone marrow stromal cell antigen 2 (BST2), also known as tetherin, HM1.24 or CD317 represents a type 2 integral membrane protein, which has been described to restrict the production of some enveloped viruses by inhibiting the virus release from the cell surface. This innate antiviral mechanism is counteracted by the HIV-1 viral factor Vpu, targeting BST2 for cellular degradation. Since antiviral BST2 activity has been mainly confirmed by in vitro data, we investigated its role in vivo on the disease progression using the SIV/macaque model for AIDS. We determined BST2 expression in PBMC and leukocyte subsets of uninfected and SIV-infected rhesus macaques by real-time PCR and flow cytometry and correlated it with disease progression and viral load.

**Results:**

Compared to pre-infection levels, we found increased BST2 expression in PBMC, purified CD4^+^ lymphocytes and CD14^+^ monocytes of SIV-infected animals, which correlated with viral load. Highest BST2 levels were found in progressors and lowest levels comparable to uninfected macaques were observed in long-term non-progressors (LTNPs). During acute viremia, BST2 mRNA increased in parallel with MX1, a prototype interferon-stimulated gene. This association was maintained during the whole disease course.

**Conclusion:**

The detected relationship between BST2 expression and viral load as well as with MX1 indicate a common regulation by the interferon response and suggest rather limited influence of BST2 in vivo on the disease outcome.

**Electronic supplementary material:**

The online version of this article (doi:10.1186/s12977-015-0219-8) contains supplementary material, which is available to authorized users.

## Methods

### Ethics statement

Rhesus macaques (*Macaca mulatta*) of Indian origin of either sex were housed at the German Primate Centre under conditions according to the German Animal Welfare act complying with the European Union guidelines on the use of non-human primates for biomedical research. All animals studies were approved by an external ethics committee authorized by the Lower Saxony State Office for Consumer Protection and Food Safety (project licenses 509.42502/08-04.03, 33.14-4502-04-017/07, 33.14.42502-04-072/08 and 33.14-42502-04-10/0037 issued by the same State Office. Rhesus macaques were kept under permanent medical supervision by veterinarians and animal caretakers. This includes measures of animal welfare and amelioration of suffering such as a 12:12 light dark schedule, a temperature between 18 and 23 °C with a humidity range of 50–60 % and provision with fresh air. Macaques were fed twice a day with dry food supplemented with fresh fruits and constant water access was provided. Environmental enhancement was realized by putting rings and perches into the cages, by task-oriented feeding methods (e.g. treats, vegetables or fruits frozen in ice cubes, food puzzle), and by playing music. In cases of suffering, macaques were euthanized in accordance to a scoring system describing termination criteria as approved by the external ethics committee and corresponding to the IACUC endpoint guidelines. The scoring system considers weight loss, defecation anomalies, water and food consumption, behavior (attention/alertness, movement disorders), respiration, size of lymph nodes, and the hemogram. Animals were euthanized by an overdose of Pentobarbital-Natrium (Narcoren^®^, Merial, Hallbergmoos, Germany) under anesthesia.

### Viral challenge and sample collection

For virus inoculation, animals received a deep anesthesia by intramuscular injection (i.m.) of a mixture of ketamine, xylazine, and atropine. Animals were challenged either tonsillar or intravenously (i.v.) with SIVmac239 [1] or intrarectally (i.r.) or intravenously (i.v.) with a SIVmac251-derived virus stock [1, 2], both prepared in rhesus monkey peripheral blood mononuclear cells (PBMC). Blood samples were collected of monkeys, sedated i.m. with 10 mg ketamine per kg body weight.

### Animals

A total of 153 animals involved in different experiments were used for this study. For whole blood analysis, blood was taken from 17 macaques before and 24 weeks after low-dose i.r. infection with SIVmac251. For comparison between relative BST2 mRNA expression and BST2 protein expression, 32 animals infected with SIVmac251 either via i.r. or i.v. routes were investigated. Another 35 infected macaques (20 with SIVmac251 and 15 with SIVmac239) were grouped according to their clinical stage in progressors and LTNPs. The 26 progressor animals displayed viral loads above 1E + 04 RNA copies/ml plasma and up to 18 progressor animals were investigated between 11 and 148 wpi when clinically still asymptomatic. Of them, 13 monkeys were euthanized and analysed between 22 and 138 wpi when first signs of AIDS appeared, as judged from clinical as well as necropsy and histopathological findings, i.e. anorexia, incurable diarrhoea, *Pneumocystis jirovecii* infection or neurological dysfunction. Another nine monkeys survived for more than 3 years post infection in the absence of any signs of immunodeficiency with a viral load below 1E + 04 RNA copies/ml and were termed as LTNPs. In a longitudinal study, two animals were inoculated intravenously with 100 TCID50, three animals with 10 TCID50 and two animals with 1 TCID50 of SIVmac251 as part of an in vivo titration experiment aimed at defining the in vivo infective dose of a new monkey PBMC-derived virus stock. This SIVmac251 challenge virus was prepared on PHA-stimulated PBMC from several monkeys. Supernatants were harvested and pooled. After filtration, aliquots were prepared and stored at liquid nitrogen and the TCID50 was determined on C8166 cells.

### Lymphocyte isolation

Peripheral blood was obtained by venipuncture and peripheral blood mononuclear cells (PBMC) were isolated via ficoll-paque gradient centrifugation (lymphocyte separation medium, PAA laboratories, Pasching, Austria). CD4^+^ T-cells and CD14^+^ monocytes were enriched from fresh PBMC by positive selection using magnetic beads (Miltenyi Biotec, Bergisch-Gladbach, Germany) and monoclonal antibodies to either CD4 or CD14. Purity of isolated CD4^+^ T-cells and CD14^+^ monocytes was assessed by flow cytometry. Only CD4^+^ T-cells and CD14^+^ monocytes with purity above 90 % were used for downstream applications.

### Flow cytometric analysis of BST2 surface expression

Surface BST2 expression was detected on different subsets of leukocytes, defined by anti-CD3-Alexa700, anti-CD4-Pacific Blue, anti-CD8-V500, anti-CD14-PerCP (all from BD Biosciences, Heidelberg, Germany) and anti-CD45-FITC (Miltenyi, Bergisch-Gladbach, Germany), by using an anti-BST2-APC conjugated antibody (RS38E, Biolegend). Labeled cells were fixed with 3 % formalin and analyzed on a BD LSRII flow cytometer (BD Biosciences, Heidelberg, Germany). The data files were evaluated using FlowJo Version 8.7 (Tree Star, Ashland, USA). Median fluorescence intensity (MFI) for granulocytes, monocytes and lymphocytes were determined.

### Induction of BST2 with type I interferon

PBMC from three healthy animals were stimulated with 10, 50 and 100 ng Human Interferon Alpha A (Alpha 2a) (PBL Biomedical Laboratories) for 16 h. Fold induction of BST2 mRNA expression was calculated using GAPDH as internal control.

### Quantification of plasma IFN-alpha

Blood samples were obtained from 18 uninfected rhesus macaques 24 h after intramuscular inoculation with 10^11^ particles of a replication incompetent adenoviral vector construct or 10^9^ PFU of a Fowlpox construct. Plasma IFN-alpha levels were quantified by ELISA using pan-specific antibodies for IFN-alpha (Mabtech, Nacka Strand, Sweden). Briefly, plasma was added to high binding microtiter plates (Greiner Bio-One GmbH, Frickenhausen, Germany) that were previously coated with respective antibodies. Bound interferon from plasma samples were detected by rabbit anti-monkey horseradish peroxidase (HRP)-conjugated sera and tetramethylbenzidine substrate (Sigma). Absorbance was measured at 405 nm with a 550 microplate reader (Bio-Rad Laboratories, Hercules, CA, USA). The detection limit of the ELISA was 10 pg/ml.

### RNA isolation and cDNA synthesis

Total cellular RNA was isolated from 2 × 10^6^–5 × 10^6^ cells with RNeasy Plus Mini Kit (Qiagen, Hilden, Germany), except for whole blood samples, where RNA was isolated with PAXgene Blood RNA Kit (Qiagen, Hilden, Germany). Purified RNA was quantified by measuring the optical density at 260 nm (OD_260_). All samples showed an OD_260_/OD_280_ ratio of 1.9 or above. RNA quality was randomly checked by Agilent 2100 Bioanalyzer (Agilent Technologies, Böblingen, Germany) showing RIN values (RNA Integrity Number) of at least 8.0. Synthesis of cDNA was performed using SuperScript III First-Strand Synthesis System for RT-PCR kit (Invitrogen GmbH, Karlsruhe, Germany) according to the manufacturer protocol.

### Quantification of BST2 and MX1 mRNA

Quantification of BST2 and MX1 mRNA levels in leukocytes were performed by real-time PCR assay using SYBR Green (Qiagen, Hilden, Germany) chemistry with primers designed to uniquely amplify BST2 (Genbank accession number NM_001161666) and MX1 (Genbank accession number EF101561). Following primers (Sigma, Hamburg, Germany) were used: BST2 forward, 5′-GACGAAAGAAAGTGGAGGAGCTT-3′ (nt 335–57); BST2 reverse, 5′-TCTCTTCTCAGTCGCTCCACCT-3′ (nt 428–407); MX1 forward, 5′-AGGAGTTGCCCTTCCCAGA-3′ (nt 295–313); MX1 reverse, 5′-TCGTTCACAAGTTTCTTCAGTTTCA-3′ (nt 372–348). MX1 primers were taken from Abel [3]. Glyceraldehyde-3-phosphate dehydrogenase (GAPDH), Genbank accession number XM_001105471) was used as a house keeping gene with following primers taken from Rodriguez-Jimenez [4] forward, 5′-CCTGCACCACCAACTGCTTA-3′ (nt 525–544) and reverse 5′-CATGAGTCCTTCCACGATACCA-3′ (nt 598–577). PCR reactions were performed in Micro Amp optical tubes or plates (Applied Biosystems GmbH, Darmstadt). Each 25 µl reaction mixture contained 12.5 µl 2 × QuantiTect SYBR Green PCR master mix (Qiagen, Hilden, Germany), 1 µl of each 10 µM primer, and 2 µl cDNA products. Samples were amplified by TaqMan-based real-time PCR on an ABI Prism 7500 sequence detection system (Applied Biosystems GmbH, Darmstadt) with one cycle at 95 °C (15 min) followed by 40 cycles at 95 °C (15 s) and 55 °C (1 min). The calculated efficiency for all primers was determined by dilution experiments and was from 97 to 98 %, thus target sequences were amplified with similar efficiency. All samples were run at least in duplicates. Results were analysed by Sequence Detection Software (Applied Biosystems GmbH, Darmstadt). Relative mRNA expression levels were calculated by using the comparative cycle threshold (Ct) method. BST2 and MX1 gene expression were normalized to endogenous control GAPDH mRNA. Relative mRNA expression levels were determined using the formula: 100 × 2^−ΔCt^. Fold induction (n-fold of baseline) was calculated by normalization to individual pre-values.

### Quantification of plasma viral RNA

Isolation of viral RNA was performed from plasma samples according to the MagAttract Virus Mini M48 protocol (Qiagen, Hilden, Germany). Purified SIV RNA was quantified with TaqMan-based real-time PCR on an ABI-Prism 7500 sequence detection system (Applied Biosystems GmbH, Darmstadt) as described [5]. Amplified viral RNA was calculated as SIV-RNA copies per millilitre plasma.

### Statistics

The statistical analyses were done with GraphPad Prism version 5.00 for Windows, GraphPad Software, San Diego, CA, USA. For multiple comparisons Kruskal–Wallis test with Dunn’s correction was used. For comparative interpretation between two groups the nonparametric two-tailed Mann–Whitney’s U test was used. For correlation the nonparametric two tailed Spearman test was performed. p values below 0.05 were regarded as significant.

## Background

Mammalian cells encode a variety of proteins capable of interfering with retroviral replication and causing intrinsic inhibition of retroviral infection [6]. BST2/CD317/HM1.24 has recently been described as one of these restriction factors, also named tetherin because of its ability to directly tether HIV-1 virions to infected cell membranes and thus potently inhibiting the release of budding virions [7–9]. Tethered particles are internalized and targeted for lysosomal [10, 11] or proteasomal degradation [12]. Consistent with a tethering mechanism, immuno-EM and biochemical studies identified BST2 on cell surface tethered virions as well as on particles tethered to each other [9, 13]. Biochemical analyses further demonstrated that BST2 forms cysteine-linked dimers modified by N-linked glycosylation [14–16].

BST2, originally identified as a membrane protein in terminally differentiated human B cells in patients with multiple myeloma [17, 18], represents a 28–36-kDa glycosylated unusual type II membrane protein. The protein is composed of an N-terminal cytoplasmatic tail, a single transmembrane spanning region, an extracellular coiled-coil domain and a C-terminal glycosyl-phosphatidylinositol (GPI) anchor incorporated in cholesterol-rich lipid rafts. BST2 is continuously cycling between the cell surface and internal membranes, including the trans-Golgi network [15, 16, 19, 20].

BST2 is expressed on a wide variety of cell types [17, 21] and has been described to potentially restrict the release of many enveloped viruses from the surface of infected cells, including members of the retrovirus, arenavirus, filovirus, herpesvirus and flavivirus families [22–26]. In order to circumvent BST2-mediated antiviral restriction, many viruses express BST2 antagonists [7, 8, 22, 27, 28]. Human immunodeficiency virus type 1 (HIV-1) Vpu and HIV-2 Env counteract human BST2 [29–32]. Most simian immunodeficiency viruses (SIVs) do not encode Vpu and use Nef to antagonize BST2 [27, 28, 33].

The BST2 promotor contains an interferon regulatory factor (IRF) binding site and it is well established that BST2 expression is strongly induced by type I interferons (IFNs) [34–37]. BST2 was also linked with innate viral sensing. Some studies demonstrate NFkappaB activation after BST2 cross-linking resulting in proinflammmatory immune responses [38–40]. Furthermore, BST2 binds to immunoglobulin-like transcript 7 (ILT7) expressed on pDCs supposedly acting as a negative regulator of interferon production by these cells [41, 42]. These findings were questioned by recent studies showing that activated mature pDCs downregulate ILT7 in vitro, indicating BST2-ILT7 cross-linking on immature circulating pDCs may function as a homeostatic regulatory mechanism rather than a negative feedback for activated mature pDCs [43].

Only little is known about the impact of BST2 during retroviral disease course. In this study, we investigated the role of BST2 in the SIV rhesus macaque model, which represents currently the best animal model to study HIV infection and AIDS pathogenesis [44, 45]. BST2 mRNA transcription levels were quantified in cross-sectional and longitudinal analyses and its surface protein expression was determined in PBMC and in SIV target cells, CD4^+^ T lymphocytes and CD14^+^ monocytes. Our results show increased BST2 expression in SIV-infected progressors animals, whereas in long term non progressors (LTNPs) only low BST2 levels were detected. A direct correlation between BST2 expression and viral load as well as with type I interferon response as evidenced by MX1 mRNA expression was found. Our data suggest that BST2 is part of the antiviral interferon response but has a limited influence on disease progression.

## Results

### BST2 is increased in SIV-infected macaques and correlates with viral load

**Fig. 1:**
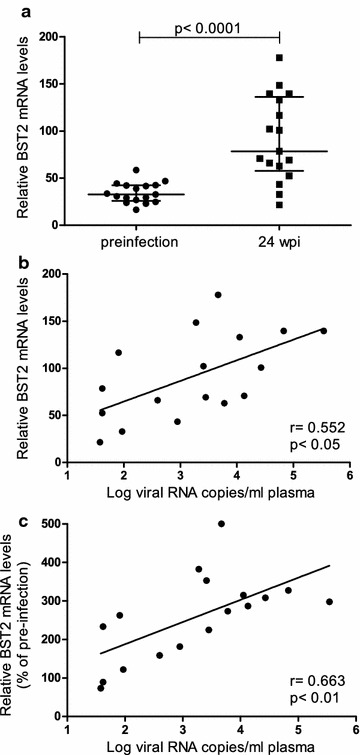
Whole blood BST2 mRNA levels in SIV-infected macaques. BST2 mRNA levels in whole blood of 17 rhesus macaques were determined using PAXgene Blood RNA Kit and real time PCR before and at 24 weeks after infection (wpi). **a** BST2 mRNA levels were compared between uninfected (*circles*) and SIVmac251 infected animals (*squares*). BST2 mRNA levels are shown in copy numbers per 100 copies of GAPDH. Horizontal lines within the clusters are depicting the median. Group comparisons were calculated using two-tailed Mann–Whitney’s U test. BST2 mRNA levels at 24 wpi (**b**) and BST2 mRNA levels after normalization to individual pre-infection values (**c**) were correlated with plasma viral load. Viral load is displayed as log-transformed RNA copies per millilitre (ml) plasma. *r* Spearman’s correlation coefficient; *regression line* is shown; *p*, p value. Each data point represents one individual animal

In order to investigate the impact of SIV infection on BST2 transcription, relative BST2 mRNA levels in whole blood of 17 rhesus macaques were determined before and 24 weeks after infection with SIVmac251 using an intrarectal (i.r.) repeated low dose inoculation scheme. RNA was isolated using the PAXgene Blood RNA Kit and BST2 mRNA was quantified by real time PCR relative to the housekeeping gene GAPDH. Relative BST2 mRNA levels were significantly increased after infection (Fig. 1a, p < 0.0001). Compared to individual pre-infection levels, the relative BST2 mRNA levels at 24 weeks post infection (wpi) were on average about threefold higher. Higher BST2 values in infected animals were significantly associated with higher plasma viral loads at 24 wpi (Fig. 1b, p < 0.05). Interestingly, this correlation was even better after normalizing relative BST2 mRNA expression levels to their individual pre-infection values (Fig. 1c, p < 0.01).

**Fig. 2:**
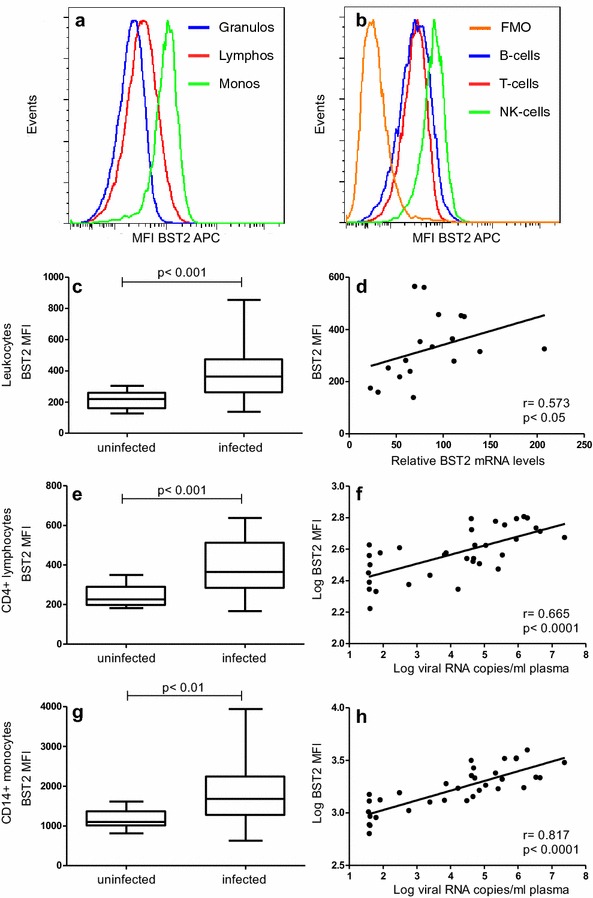
BST2 mRNA and protein expression in uninfected and SIVmac251-infected macaques. Representative flow cytometric analyses of BST2 surface expression of an uninfected animal shown as histogram for leukocyte populations (**a**) and histogram for lymphocyte subsets including a fluorescence minus one (FMO) control (**b**). BST2 surface expression, displayed as median fluorescence intensity (MFI) on all leukocytes (**c**), on CD4^+^ lymphocytes (**e**) and on CD14^+^ monocytes (**g**) is illustrated for 12 uninfected and 32 SIVmac251 infected animals at 24 wpi. *Box plots* depict median and quartiles while whiskers show range. Group comparisons were calculated using two-tailed Mann–Whitney’s U test. **d** Whole blood BST2 mRNA levels correlate with BST2 surface protein expression. BST2 mRNA levels are depicted as copy numbers per 100 copies of GAPDH. BST2 surface expression on CD4^+^ lymphocytes (**f**) and CD14^+^ monocytes (**h**) correlates with plasma viral load. Each data point represents one individual animal. *Regression lines* are depicted; *r* Spearman’s correlation coefficient; *p*, p value

We next investigated whether the increased relative BST2 mRNA levels 24 weeks after infection also translated into increased BST2 protein expression. To this end, 32 SIV-infected monkeys from two additional experiments were analysed. Twenty animals had been repeatedly infected i.r. with low doses of SIVmac251, another 12 monkeys were infected intravenously (i.v.) with 100 TCID50 of the same viral stock. Again, BST2 mRNA levels were significantly increased compared to pre-infection levels (p < 0.01) and correlated significantly with viral load (p < 0.05) (data not shown). Surface expression of BST2 was then determined in these monkeys by flow cytometry and compared with the one of twelve uninfected monkeys analysed the same day. Surface expression of BST2, shown as median fluorescence intensity (MFI) of allophycocyanin (APC), differs in uninfected animals between major leukocyte subsets (Fig. 2a; MFI granulocytes 179 ± 16.5, monocytes 1164 ± 87, lymphocytes 338 ± 30). Among lymphocyte subsets, B-cells (MFI 360 ± 44) and T-cells (MFI 308 ± 22) showed similar BST2 expression, whereas NK-cells (MFI 822 ± 64) showed the highest expression (Fig. 2b and Additional file 1: Figure S1A).

Surface expression of BST2 on total leukocytes was significantly increased in blood of infected animals compared to uninfected monkeys (Fig. 2c, p < 0.001). Moreover, protein expression correlated well with the respective BST2 mRNA levels in SIV-infected monkeys (Fig. 2d, p < 0.05). A similar picture was seen when investigating BST2 expression in CD4^+^ lymphocytes and monocytes, the major target cells of SIV. Surface expression of BST2 was significantly higher in infected monkeys than in uninfected controls (Fig. 2e, p < 0.001 and Fig. 2g, p < 0.01) and BST2 expression on these cell populations correlated significantly with plasma viral load (Fig. 2f, p < 0.0001 and H, p < 0.0001). Despite different normal levels in monocytes and CD4^+^ lymphocytes, the relative increase was similar in both leukocyte populations.

Also for other leukocyte populations, granulocytes, B cells and CD8 + T cells, increased BST2 levels and correlations between BST2 expression and plasma viral load were observed (Additional file 1: Figure S1A–H). NK cells showed also higher BST2 expression but no correlation with viral load was seen (Additional file 1: Figure S1I and J). In summary, SIV infection resulted in increased levels of BST2 in all leukocyte subsets tested, which correlated with SIV RNA copy numbers.

### BST2 is regulated by type I interferons in vitro and in vivo

**Fig. 3:**
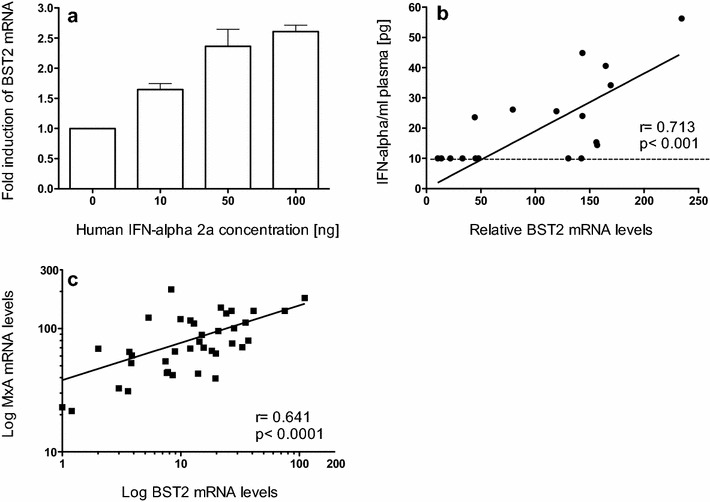
BST2 mRNA induction by type I interferon. **a** Fold induction of relative BST2 mRNA in PBMC from three uninfected rhesus macaques after stimulation with human Interferon Alpha A (Alpha 2a) for 16 h. Data are expressed as fold increase over baseline after normalization to pre-treatment values. *Error bars* represent standard deviation, **b** relative mRNA copies of BST2 in PBMC (shown in copy numbers per 100 copies of GAPDH) are illustrated in relation to plasma IFN-alpha levels from blood samples of 18 uninfected rhesus macaques 24 h after inoculation of replication incompetent adenovirus or fowl pox vectors. The black dashed line indicates the detection limit of the ELISA and **c** whole blood MX1 mRNA levels correlate with BST2 mRNA determined in 38 SIVmac251 infected rhesus macaques at 24 wpi. Relative mRNA levels are depicted as log-transformed copy numbers per 100 copies of GAPDH. Each data point represents one animal. *Regression line* is shown; *r*, Spearman’s correlation coefficient; *p*, p value

Human BST2 expression can be influenced by type I interferons in vitro and in vivo [34–37]. Similarly, a dose-dependent increase in BST2 transcription by IFN-alpha in macaque PBMC in vitro was found (Fig. 3a). Furthermore, a direct correlation between BST2 transcription levels and in vivo plasma interferon-alpha levels exists (Fig. 3b). However, the application of the IFN-alpha assay is restricted due to its low sensitivity. Therefore, the influence of type I interferons on BST2 expression in vivo was assessed by quantifying the transcription levels of MX1, which has long been used as a reliable marker for type I interferon bioactivity, but may be influenced by other cytokines as well. We speculated that the increased levels of BST2 found in infected animals were associated with the interferon response known to be elevated in these animals [46, 47]. MX1 levels were assessed in a total of 38 monkeys at 24 wpi. Indeed, MX1 levels in whole blood correlated well with BST2 (Fig. 3c, p < 0.0001) indicating BST2 can be regulated by type I interferons in vivo.

### BST2 is increased in progressors but not in LTNPs

To evaluate the role of BST2 on disease course, we used stored material from previous experiments where survival times of SIV-infected monkeys were known. To this end, 35 animals infected intravenously (20 with SIVmac251, 15 with SIVmac239) were grouped according to their survival time and viral load. A total of 26 macaques displayed a progressive course of infection with viral load above 1 × 10^4^ copies/ml. Among them, 13 animals had developed AIDS-like symptoms at time samples were analysed. Another nine monkeys, which had survived for more than 3 years post infection in the absence of any signs of immunodeficiency and with a viral load below 1 × 10^4^ copies/ml were termed LTNPs. Samples obtained from nine uninfected animals during the same time frame, were used as negative control group.

**Fig. 4:**
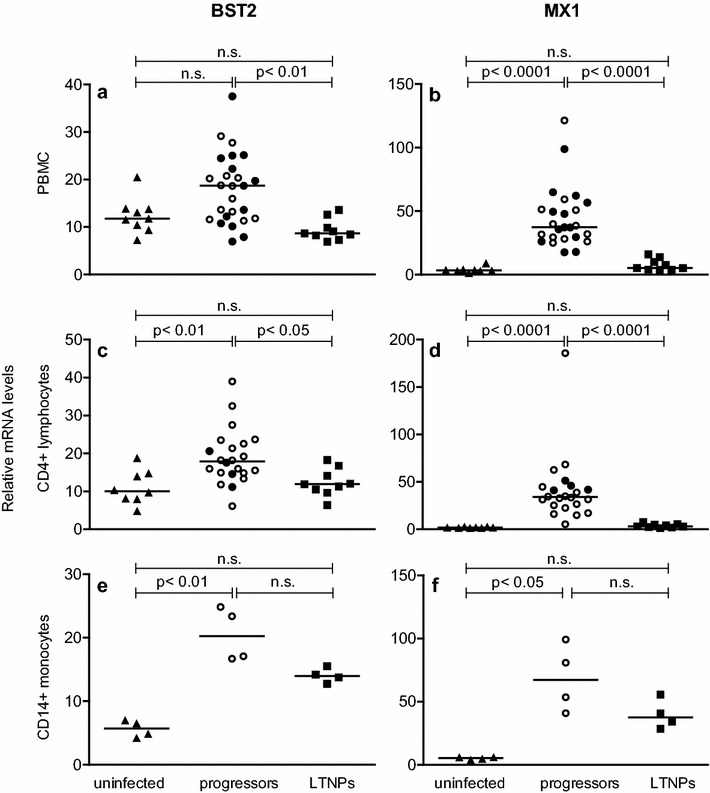
BST2 and MX1 mRNA levels in uninfected and SIV-infected macaques with different disease progression. Relative BST2 (*left panels*) and relative MX1 (*right panels*) mRNA levels were determined in uninfected (triangles), SIV-infected asymptomatic progressors (*empty circles*), progressors with AIDS-like symptoms (filled *circles*) and LTNPs (*squares*). Relative BST2 and MX1 mRNA levels are shown in copy numbers per 100 copies of GAPDH in PBMC (**a**, **b**), CD4^+^ lymphocytes (**c**, **d**) and CD14^+^ monocytes (**e**, **f**). Each data point represents one individual animal. *Horizontal lines* within the clusters are depicting the median. Group comparisons were calculated using Kruskal–Wallis test with Dunn’s multiple comparison analysis; *p*, p value; *ns* not significant

In this experiment, RNA was isolated from Ficoll-separated PBMC. Similar to the first experiments where whole blood was investigated, infected animals with progressive disease showed increased BST2 mRNA levels in PBMC. However this was statistically not significant (Fig. 4a, p = 0.052). No differences in BST2 or MX1 expression levels were observed between asymptomatic progressive animals (Fig. 4a–d; empty circles) and those with AIDS-like symptoms (Fig. 4a–d; filled circles). BST2 RNA levels were comparable between SIVmac239- and SIVmac251-infected macaques (data not shown). As in the previous experiments, BST2 levels correlated significantly with viral load (data not shown). Moreover, analysis of magnetically purified target populations for SIV revealed significantly higher BST2 mRNA levels also in CD4^+^ lymphocytes (Fig. 4c) and in CD14^+^ monocytes of progressor animals (Fig. 4e), corroborating the flow cytometric data and demonstrating for the first time a correlation between BST2 expression and disease progression in SIV-infected macaques.

In LTNPs however, BST2 levels were comparable to uninfected monkeys both in bulk PBMC as well as in purified CD4^+^ lymphocytes (Fig. 4a, c). Only for CD14^+^ monocytes, a trend to higher BST2 levels in LTNPs was observed, not reaching statistical significance (Fig. 4e). BST2 levels (Fig. 4, left panels) were in good accordance with MX1 levels (Fig. 4, right panels) in all cell populations and disease progression groups, again supporting the notion that BST2 is mainly regulated by type I interferons in vivo.

### Kinetics of BST2 transcription during early infection

**Fig. 5:**
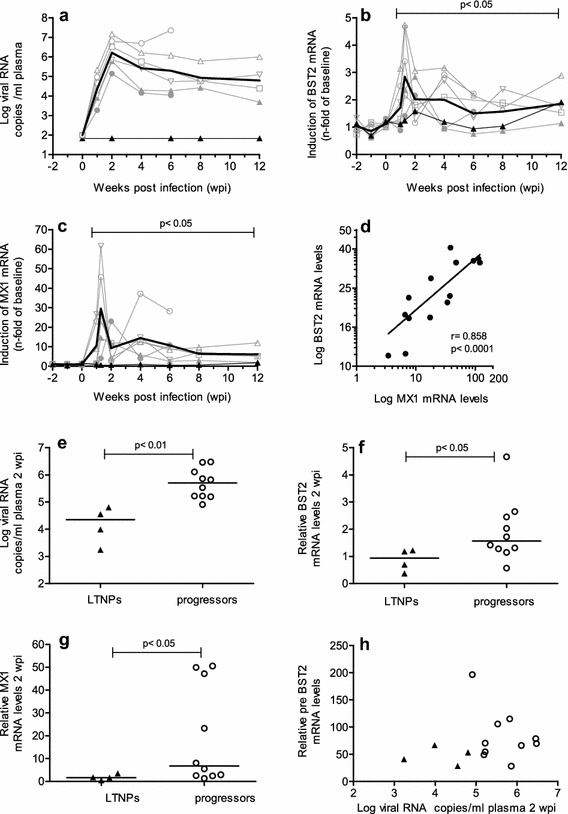
Kinetics of RNA plasma viral load and mRNA levels of BST2 and MX1 in SIV-infected macaques. Plasma viral load as well as relative BST2 and MX1 mRNA levels in PBMC were determined longitudinally in seven macaques before and after inoculation with SIVmac251 MPBMC (**a**–**c**). Viral load is depicted as log-transformed RNA copies per millilitre (ml) plasma (**a**). Relative BST2 mRNA (**b**) and relative MX1 mRNA (**c**) in PBMC were calculated as copy numbers per 100 copies of GAPDH. Data are expressed as fold increase over baseline after normalization to the mean of three pre-infection values. Fine *grey lines* with *symbols* represent individual infected animals. Fine *black lines* with *closed triangles* depict the one animal inoculated but not infected. *Bold lines* show mean values of infected animals. p value show a significant difference to the mean of the three pre-infection values calculated by Mann–Whitney’s U test. **d** Relationship between relative BST2 and MX1 mRNA levels in PBMC from rhesus macaques during acute SIV infection (10–14 dpi). *Line* shows linear regression; *r*, Spearman’s correlation coefficient; *p*, p value. Samples from LTNPs (*filled triangles*) and progressor (*open circles*) rhesus macaques were analysed for viral load (**e**), relative BST2 (**f**) and relative MX1 (**g**) RNA levels at 2 wpi. **h** Relationship between pre-infection BST2 mRNA levels and viral load at 2 wpi. Each data point represents one individual animal. *Horizontal lines* within the clusters are depicting the median. Group comparisons were calculated using Mann–Whitney’s U test

Our results from the cross-sectional studies suggest an induction of BST2 together with MX1 during the asymptomatic phase of infection. In order to determine more precisely the kinetics upon infection, BST2 and MX1 transcription were assessed during the 1st weeks of infection in seven animals infected with different doses of SIVmac 251MPBMC as part of an in vivo titration study. All animals, except one of those inoculated with the lowest dose, became infected and showed a typical course of plasma viral load (Fig. 5a). This experiment was terminated early after infection and macaques were euthanized at predetermined time points between six and 30 wpi without signs of AIDS. Figure 5 shows the kinetics of BST2 (B) and MX1 transcription (C) for PBMC in these macaques normalized to the mean of three independently measured individual pre-infection values. The inoculated macaque that remained uninfected served as control. One week after infection, we observed a simultaneous increase of BST2 and MX1 transcripts in PBMC compared to pre-infection values, which reached a maximum at 10 days post infection (dpi) (Fig. 5b, c). This happened shortly before peak viremia, which occurred 2 weeks after infection (Fig. 5a). In the single animal, which remained uninfected after inoculation, these variations were not seen. Later in the course of infection the transcription of BST2 and MX1 remained significantly elevated above pre-infection values (Fig. 5b, p < 0.05 and Fig. 5c, p < 0.05). This parallel kinetics of BST2 and MX1 expression, and the similar transcription patterns of both genes analysed cross-sectionally (Fig. 4) suggest common regulatory mechanisms for BST2 and MX1 already during acute SIV infection. Indeed, BST2 and MX1 mRNA expression levels are directly correlated at ten to 14 dpi in PBMC (Fig. 5d, p < 0.0001).

Furthermore, four animals that became LTNPs and 10 macaques that displayed progressive disease course were compared with respect to their viral load, relative BST2 and relative MX1 mRNA expression levels at acute SIV infection (Fig. 5e–g). Future LTNPs showed significantly lower BST2 and MX1 mRNA expression levels at 2 wpi (Fig. 5f, g) than the future progressors, probably attributable to reduced viral loads (Fig. 5e). These differences in BST2 expression between LTNPs and progressors were preserved later during chronic infection. Moreover, no relationship between relative pre-infection BST2 mRNA levels and viral load at 2 wpi was observed (Fig. 5h). In summary, individual differences in BST2 levels before infection do not influence early viral replication and higher levels of BST2 during peak viremia are not associated with a favourable disease course.

## Discussion

The present study investigated the role of BST2 during the natural course of retroviral infection in the SIV/macaque model for AIDS. In line with previous studies using human cells [35, 48, 49], we found surface expression of BST2 on all blood leukocyte populations, with highest levels on CD14^+^ monocytes [50]. Compared to uninfected animals, BST2 was increased in SIV-infected rhesus macaques both at the RNA and the protein level in all leukocyte subsets investigated, including CD4^+^ T lymphocytes and CD14^+^ monocytes, the major target cells of SIV infection (compare Figs. 2, 4). Longitudinal analyses revealed that BST2 levels steeply increase following i.v. infection to reach a maximum shortly before peak viremia, later dropping to levels still higher than before infection, corroborating a recent study where macaques were infected by the rectal route with a lymphotropic viral clone [50]. Similarly, higher levels of BST2 were found in HIV-infected patients within the first 4 weeks of infection compared to chronic infection [35]. In extension to these previous studies, we were also able to study for the first time the relationship between BST2 levels and disease course by combining data from several infection experiments. In all experiments and at different phases of infection, BST2 transcription but also protein expression was positively correlated with plasma viral load. Although, compared to acute viremia, low BST2 levels in the chronic phase, and even lower BST2 levels in patients under ART, were observed before, such a direct correlation between viral load and BST2 levels has not been reported yet, probably due to the small number of individuals studied [35, 36, 50]. Interestingly, BST2 levels were significantly lower in LTNPs than in progressors but in the same range as in uninfected monkeys, reminiscent to HIV-infected patients after antiretroviral therapy [35].

BST2 has not only been shown to counteract viral replication in vitro, its importance in vivo is demonstrated by several lines of evidence for a strong selective pressure on immunodeficiency viruses to inhibit BST2. For example the switch from Nef as simian BST2 to Vpu as human BST2 antagonist is thought to present an important step during the evolution of HIV from a zoonosis to a pandemic virus [33]. Vice versa recent success in adapting HIV to macaques was associated with mutations rendering vpu capable of antagonizing macaque BST2 [51]. Similarly earlier studies demonstrated that some HIV Vpu proteins are also able to inhibit rhesus BST2 to some extent and that Vpu-deficient SHIVs are less pathogenic in vivo [52]. Moreover, by extensively analyzing Vpu variants isolated from several HIV-patients at different time points of infection it was recently shown that the function of Vpu remains preserved despite high variability even within an individual, indicating that BST2 function has to be kept at bay during acute and chronic stages of infection [53]. In our model, rhesus BST2 is antagonized by SIVmac Nef [27, 28, 54]. So far selective pressure on Nef activity against BST2 has not been demonstrated, and although BST2 antagonism is genetically separable from other Nef functions [54] the pathogenicity of such mutants has not been assessed in vivo. As BST2 has been described to prevent virion release [7–9] and in the light of the strong selective pressure on the virus to counteract BST2 activity, higher BST2 expression should result in lower plasma viral RNA levels. This was not the case in our study suggesting that the antagonistic effect of SIV was sufficient to even counteract modestly increased BST2 levels. Along this line, it was shown that anti BST2 activity was not different between variants isolated from HIV-infected patients with either rapid or LTNP disease course [53].

On the other hand, data generated from BST2 deficient mice show that the antiviral effect of BST2 may be more limited in vivo [55]. Moreover, there is growing evidence that BST2 does not actually restrict HIV-1 replication but simply shifts virus spread from a cell-free to a cell-to-cell transmission mode [56–59]. Such a cell-to-cell spread of HIV-1 has been described as an efficient viral dissemination mechanism, being more rapid [60, 61], and even interferon insensitive [62]. This suggests that, once the infection is established, increased BST2 expression may favour viral dissemination by escaping host immune response [63].

BST2 is regulated by exogenous cues like type I interferons and intrinsic signal cascades after sensing infections through TLRs, both convening at transactivation of BST2 through IRFs [37, 48, 64]. As BST2 was uniformly upregulated on all leukocytes and not only on infected cells it is clear that soluble factors are responsible for the increased levels in SIV-infected macaques. In vitro, BST2 transcription seems to be specifically controlled by IFN-alpha as other cytokines such as IL-6, TNF-alpha did not influence BST2 expression [35]. Thus it comes as no surprise that our results showed a strong correlation throughout different phases of the infection between BST2 levels and MX1, a prototype interferon stimulated gene (ISG), complementing a previous study, which demonstrated similar kinetics of BST2 expression and IFN-alpha levels in plasma in the acute phase but did not find a correlation between the two parameters [50]. One possible reason for this discrepancy is that IFN-alpha in plasma becomes undetectable by ELISA in the chronic phase of the infection and is therefore less sensitive for determining the interferon response [65] than measuring ISG like MX1 as in our work or ISG15 [36]. In agreement with results from present study, Aamer and colleagues also observed a maximum BST2 and MX1 expression during acute infection [66]. Strong induction of BST2 together with other restriction factors has also been observed in vivo after treatment of HIV/HCV-coinfected patients with pegylated IFN-alpha/ribavirin (IFN-alpha/riba) exhibiting a good correlation with ISG15 [36]. The only other cytokine known to directly enhance BST2 transcription is IL-27 [49]. It is not yet known whether this cytokine plays a role in vivo as there are conflicting data on IL-27 levels in HIV patients [43, 51]. Because IL-27 also stimulates MX1 transcription [67] it is not possible to differentiate between the effects of IFN-alpha and IL-27 in our study.

Considering the clear association between viral load and BST2 levels throughout the infection and the lower expression of BST2 in LTNPs as early as 2wpi, there is little evidence that BST2 has a positive effect on disease progression in the SIV model for AIDS. This is further underlined by the fact that there was no relationship between pre-infection BST2 mRNA levels and early viral load. In addition, no impact of pre-viremia BST2 levels with either the extent of viral load reduction after peak viremia or post acute virus RNA levels in plasma was found (data not shown).

On the other hand, human BST2 also exhibits signal transduction properties leading to NFkappaB activation and secretion of proinflammatory cytokines [38] and it has been hypothesized that this may lead to a positive feedback loop perpetuating increased BST2 expression [50]. However, the signaling trait seems to be an evolutionary recent acquisition and macaque BST2 lacks this activity [38]. Thus we think that, at least in the SIV macaque model, signaling through other pattern recognition receptors is more important for keeping BST2 levels increased. In summary, its expression levels seem to just reflect the immune activation associated with the ongoing viral replication. This is in clear contrast to other restriction factors like the APOBEC3G and -3F deaminases, for which we previously reported a negative correlation with plasma viral load and higher levels in LTNPs [47]. Indeed, in those animals, where we had the opportunity to measure both restriction factors, we observed a trend for a negative correlation between APOBEC3G, APOBEC3F and BST2 (data not shown). Only during acute infection with the dominant influence of the type I interferon response both antiretroviral factors were positively correlated. This clearly demonstrates that BST2 and the APOBEC3 deaminases are regulated differently in vivo and reflects results from in vitro studies where, in addition to IFN-alpha, numerous cytokines (IL-2; IL-15 [53], IL-27 [67], IL-32 [68] affect transcription of APOBEC3 genes, while BST2 transcription is mainly stimulated by IFN and to some extent by IL-27 [49]. Deciphering the mechanisms responsible for the different regulation in vivo might help to shape the antiretroviral response by restriction factors in a favorable way.

The lack of an obvious impact of BST2 on disease progression in our study is reminiscent to Moloney murine leukemia virus (Mo-MLV) infection in mice where genetic ablation of BST2 did not alter viral load and disease course [34]. There are however several differences between the two model systems. In contrast to mice, BST2 is constitutively expressed on leukocytes of primates and Mo-MLV does not induce an interferon response whereas interferon is elevated in SIV-infected macaques. Indeed, when using a different murine leukemia virus (LP-BM5), capable of inducing an interferon response, disease progression was somewhat aggravated in BST2-deficient mice [34]. Extrapolating the relatively small effects in the complete absence of BST2, possible small differences in BST2 expression in macaques will have even less impact on SIV-infection. However, it is still possible that BST2 influences viral replication and the natural course of the infection under certain circumstances. For example during initial infection locally produced IFN may increase BST2 expression on surrounding cells and thus limit early spread of the virus. Such an effect might be most relevant in situations where the viral antagonists of BST2 function are missing, for example after infection with nef-deleted SIV, where mechanisms acting before peak viremia determine disease progression [69]. Given the low number of animals investigated during this early time point and the fact that we used blood cells to determine BST2 and ISG levels, our present study was likely not powered to detect such subtle effects. There is however recent evidence that the interferon response indeed influences very early events during infection. As an example, treatment of SIV-infected animals with a type I interferon receptor antagonist blunted the infection induced BST2 induction, led to increased viral replication during acute infection and accelerated disease progression [54]. Conversely, treatment of macaques with pegylated IFN-alpha 2a, starting 1 week before challenge and proceeded 4 weeks after systemic infection, increased BST2 levels and the number of i.r. low dose challenges necessary to achieve systemic infection [54]. As these treatments also modulated other restriction factors the contribution of BST2 to the positive effects of the interferon response remains uncertain. There is however some evidence that BST2 may indeed help to reduce viral replication in the chronic phase of HIV-infection. IFN-alpha/riba treatment of ART naïve HIV-patients increased BST2 levels and transiently reduced plasma viral load [36]. Of the interferon induced restriction factors investigated (APOBEC3G, APOBEC3F, BST2), only BST2 exhibited a significant correlation with reduction of plasma viral load. In addition, in two of nine patients mutations developed in the Vpu sequence during treatment, which were associated with enhanced down-regulation of BST2.

Taken together, variations of BST2 levels seem to only have measurable effects on viral replication at supraphysiological levels induced by pharmacological treatment. However, the observed mutations leading to increased resistance of HIV against BST2 will limit the long term effect of such a treatment. In addition, prolonged treatment with pegylated IFN-alpha 2a results in desensitization of the interferon response [54] further curtail the clinical benefit of such a treatment. In situations however, where viral replication is low for example after interruption of ART [52] BST2 together with other interferon induced mechanisms may help to hold the viral replication at bay.

## Conclusions

By detecting an association between elevated BST2 expression with plasma viral load and only low BST2 levels in LTNPs, our results indicate rather limited effects of BST2 in vivo on the disease outcome in the SIV macaque model for AIDS. During acute viremia, BST2 mRNA levels increased in parallel with the prototype interferon-stimulated gene MX1. This correlation was maintained during the entire disease course and suggests BST2 expression as a part of the antiviral interferon alpha response reflecting immune activation associated with ongoing viral replication. Future investigations are necessary to evaluate a possible role of BST2 in limiting viral spread during early SIV/HIV infection and to assess its potential impact on shifting from a cell-free to a cell-to-cell virus transmission.

## Additional file

10.1186/s12977-015-0219-8 BST2 protein expression in uninfected and SIVmac251-infected macaques. Comparison of BST2 surface expression, displayed as median fluorescence intensity (MFI) on different leukocyte populations from uninfected monkeys (A). Comparison of BST2 surface expression between uninfected animals and infected animals for granulocytes (C), on B cells (E), CD8 + T cells cells (G) and NK cells (J). Horizontal lines depict median and quartiles. Group comparisons were calculated using Kruskal–Wallis test with Dunn’s multiple comparison analysis (A) and two-tailed Mann–Whitney’s U test (C, E, G, I). Correlations of plasma viral load with BST2 surface expression on granulocytes (D), B cells (F), CD8 + T cells (H) and NK cells (J). Each data point represents one individual animal. Regression lines are depicted; r, Spearman’s correlation coefficient; p, P value.

## Abbreviations

Ad: adenoviral vector
AIDS: acquired immune deficiency syndrome
APC: allophycocyanin
APOBEC: apolipoprotein B mRNA editing enzyme, catalytic polypeptide-like
ART: antiretroviral therapy
BST2: bone marrow stromal cell antigen 2
cDNA: complementary deoxyribonucleic acid
Ct: cycle threshold
Dpi: days post infection
ELISA: enzyme-linked immunosorbent assay
Env: envelope
FITC: fluorescein isothiocyanate
GAPDH: glyceraldehyde-3-phosphate dehydrogenase
GPI: glycosyl-phosphatidylinositol
HCV: hepatitis C virus
HIV: human immunodeficiency virus
IACUC: institutional animal care and use committees
IFNs: interferons
IL: interleukin
ILT7: immunoglobulin-like transcript 7
i.m.: intramusculary
immuno-EM: immuno electron microscopy
i.r.: intrarectally
IRF: interferon regulatory factor
ISG: interferon stimulated gene
i.v.: intravenously
LP-BM5: murine leukemia virus isolate
LTNPs: long-term non-progressors
MFI: median fluorescence intensity
Ml: millilitre
Mo-MLV: moloney murine leukemia virus
MPBMC: monkey peripheral blood mononuclear cells
mRNA: messenger ribonucleic acid
MX1: myxovirus (influenza virus) resistance 1
Nef: negative regulatory factor
NFkappaB: nuclear factor kappa-light-chain-enhancer of activated B cells
NK-cells: natural killer cells
p p: value
PBMC: peripheral blood mononuclear cells
PCR: polymerase chain reaction
pDCs: plasmacytoid dendritic cells
PerCP: peridinin chlorophyll
PFU: plaque-forming units
PHA: phytohemagglutinin
R: Spearman’s correlation coefficient
SHIV: simian/human immunodeficiency virus
SIV: simian immunodeficiency virus
TCID50: 50 % tissue culture infective dose
TLRs: toll-like receptors
TNF: alpha tumor necrosis factor alpha
Vpu: viral protein unique
Wpi: weeks post infection
VSV-G: vesicular stomatitis virus G protein
