# Supplementary figures and images for: Increased BST2 expression during simian immunodeficiency virus infection is not a determinant of disease progression in rhesus monkeys
Source: Retrovirology. 2015 Nov 10;12:92. doi: 10.1186/s12977-015-0219-8 (PMC4641394; doi:10.1186/s12977-015-0219-8)

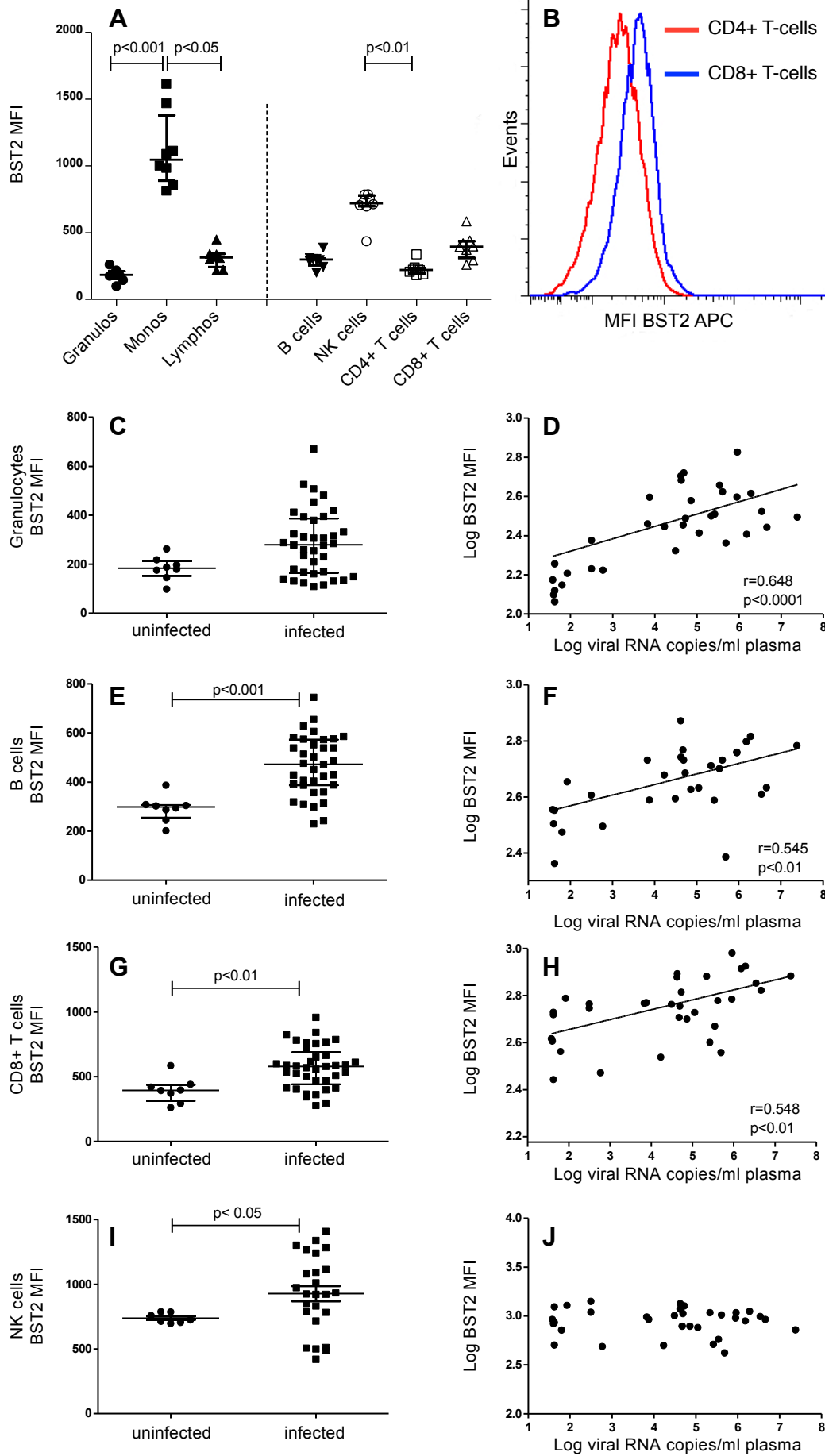

Supplement: Supplementary file 1 — 10.1186/s12977-015-0219-8 BST2 protein expression in uninfected and SIVmac251-infected macaques. Comparison of BST2 surface expression, displayed as median fluorescence intensity (MFI) on different leukocyte populations from uninfected monkeys (A). Comparison of BST2 surface expression between uninfected animals and infected animals for granulocytes (C), on B cells (E), CD8 + T cells cells (G) and NK cells (J). Horizontal lines depict median and quartiles. Group comparisons were calculated using Kruskal–Wallis test with Dunn’s multiple comparison analysis (A) and two-tailed Mann–Whitney’s U test (C, E, G, I). Correlations of plasma viral load with BST2 surface expression on granulocytes (D), B cells (F), CD8 + T cells (H) and NK cells (J). Each data point represents one individual animal. Regression lines are depicted; r, Spearman’s correlation coefficient; p, P value. [file 12977_2015_219_MOESM1_ESM.pdf]
